# Report of a Novel Mutation in *MLH1* Gene in a Hispanic Family from Puerto Rico Fulfilling Classic Amsterdam Criteria for Lynch Syndrome

**DOI:** 10.1155/2014/527946

**Published:** 2014-10-20

**Authors:** Juan M. Marqués-Lespier, Yaritza Diaz-Algorri, Maria Gonzalez-Pons, Marcia Cruz-Correa

**Affiliations:** ^1^Department of Medicine, School of Medicine, University of Puerto Rico Medical Sciences Campus, San Juan, PR 00936-5067, USA; ^2^University of Puerto Rico Comprehensive Cancer Center, P.O. Box 365067, San Juan, PR 00936-5067, USA; ^3^Departments of Medicine, Surgery and Biochemistry, School of Medicine, University of Puerto Rico Medical Sciences Campus, San Juan, PR 00936-5067, USA

## Abstract

In Puerto Rico, colorectal cancer (CRC) represents the second leading cause of cancer in men and women. Familial CRC accounts for 10–15% of the total CRC cases, while Lynch syndrome accounts for approximately 2–4% of cases. Limited information is available about the prevalence, clinical manifestations, and genetic mutations of hereditary CRC in US Hispanic individuals. In this paper we report a novel mutation in the *hMLH1* gene in a Puerto Rican Hispanic family with Lynch syndrome recruited through the Puerto Rico Familial Colorectal Cancer Registry (PURIFICAR). Our proband was identified by applying Amsterdam and Bethesda criteria for Lynch syndrome, analysis of protein expression by immunohistochemistry, and genetic sequencing of the mismatch repair genes. A novel mutation at c.2044_2045 in *hMLH1* consisting of the deletion of two consecutive nucleotides (AT) at exon 18 was identified. This deletion causes a frameshift in the protein coding sequence at p.682 resulting in premature termination and a truncated MLH1 protein. To our knowledge, this mutation has not been previously reported in the literature. The detection of this novel mutation in *MLH1* further emphasizes the need for genetic testing in at-risk patients for hereditary CRC from various ethnic and racial backgrounds.

## 1. Introduction

In Puerto Rico, colorectal cancer (CRC) represents the second leading cause of cancer in men and women and the first cause of cancer death [1]. In the US, CRC is the third leading cause of death in Hispanic population accounting for 16.1/100,000 deaths in males and 10.7/100,000 in women [2]. Familial CRC accounts for 10–15% of all CRCs [3, 4] and several studies suggest that inheritance has a significant impact in the pathogenesis of up to one third of all CRC cases [5, 6]. One such familial syndrome, accounting for about 1% of all CRC cases, is familial adenomatous polyposis (FAP) [7]. Lynch syndrome is a highly penetrant, autosomal dominant cancer-susceptibility syndrome that accounts for approximately 2–4% of cases of total CRC cases [8, 9]. Lynch syndrome is caused by germline defects on the DNA mismatch repair (MMR) mechanism caused by a mutation in one of the MMR genes [9]. Individuals with Lynch syndrome are genetically predisposed to develop malignancies and have a 40–80% lifetime risk of developing by age 75 [10–12].

Individuals with Lynch syndrome develop adenomas at the same rate as the general population, but adenomas tend to develop at an earlier age, have more villous components, and progress more rapidly [10]. Lynch syndrome is associated with tumorigenesis caused by mutations in one of several genes involved in DNA mismatch repair system (MMR) [13]. An additional somatic event in the wild-type allele is necessary to make both copies of the MMR gene inactive, which predisposes to accumulation of somatic mutations leading to carcinogenesis. Lynch syndrome mutation carriers are also at risk for other tumors including endometrial cancer and to a lesser extent other cancers such as tumors of the stomach, small bowel, ovary, upper uroepithelial tract, biliary tract, skin, and brain [10]. Specific pathological characteristics of Lynch syndrome colorectal tumors have been identified but none of them are pathognomonic. Mutations leading to the change of an amino-acid residue in a highly conserved region of the MMR proteins are usually considered to be pathogenic [14]. Alterations in the MMR genes usually result in the accumulation of replication errors, resulting in a hypermutable phenotype known as microsatellite instability (MSI), which occurs in a high percentage of Lynch syndrome tumors [9]. At present, eight MMR genes are known (*hMLH1*,* hMLH3*,* hMSH2*,* hMSH3*,* hMSH6*,* hPMS1*,* hPMS2*, and* hEPCAM*); however, the vast majority of Lynch syndrome cases (90–95%) result from germline* hMLH1* and* hMSH2* mutations [15].

Due to the limited information available on the prevalence, clinical manifestations, and genetic mutations in US Hispanic individuals with hereditary CRC, variants of uncertain significance (VUS) limit the interpretation of genetic testing results among Hispanics who undergo germline genetic testing. Published data suggests that among Hispanics the majority of MSI CRC tumors may be attributed to Lynch syndrome, since prevalence of MSI in sporadic colorectal tumors among Hispanic patients is low [2]. Using the Puerto Rico Familial Colorectal Cancer Registry (PURIFICAR; http://purificar.rcm.upr.edu/index_eng.html) we identified patients meeting Amsterdam criteria and/or Bethesda guidelines for Lynch syndrome who have had germline genetic testing [16]. The objective of this study was to report a novel MMR mutation found in a Puerto Rican Hispanic family with Lynch syndrome recruited through the Puerto Rico Familial Colorectal Cancer Registry (PURIFICAR).

## 2. Methods

### 2.1. Recruitment

The proband was identified through PURIFICAR [17]. Individuals with either personal or family history of possible familial colorectal cancer and/or polyposis are referred to PURIFICAR by gastroenterologists, oncologists, and colorectal surgeons. This registry was established in 2006 at the University of Puerto Rico Comprehensive Cancer Center (UPRCCC) and has received direct or in kind support from the National Institutes of Health, UPRCCC, the Puerto Rico Gastroenterology Association (http://www.gastropr.org/), and the Puerto Rico Colorectal Cancer Coalition (http://cancercolonpr.org/). Subjects enrolled in PURIFICAR are US Hispanic individuals with a clinical and/or genetic diagnosis of FAP, attenuated FAP (AFAP), hamartomatous polyposis syndromes, or Lynch syndrome. Consented probands complete a comprehensive baseline questionnaire capturing medical, environmental exposures and cancer family history. Pedigrees for each proband are completed to trace the number of affected relatives with polyposis and/or cancer using PROGENY software (http://www.progenygenetics.com/) [18]. Pathology reports and medical/surgical records are obtained to confirm cancer diagnosis reported by probands. Affected family members living in Puerto Rico and US are also invited to participate in the Registry. In this database, 60% of the probands identified are female with an average age of diagnosis of 46.3 (±13.39 years). The distribution of the mutations consists of the following: 37.14% in the MLH1 gene, 60% in MSH2, and 2.86% in the MSH6 gene. For the current study, analysis was limited to the proband and his family.

### 2.2. Patient History

**Figure 1:**
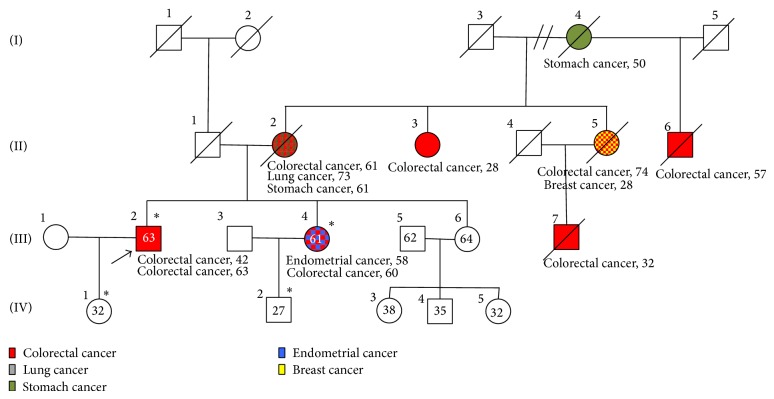
Proband's Pedigree. Pedigree of the Hispanic family from Puerto Rico fulfilling classic Amsterdam criteria for Lynch syndrome. Exon 18 novel mutation is presented as ∗.

Our proband, defined as the first person in the family in which the mutation was detected, is a 63-year-old male with a past medical history of hypertension, hypercholesterolemia, and chronic alcohol and tobacco use (23 packs a year). He presents with history of recurrent metachronous colorectal adenocarcinomas. He was first diagnosed at the age of 42 years (1989) with an apple core tumorous mass in transverse colon, which was confirmed to be a moderate-poorly differentiated adenocarcinoma. He underwent partial colon resection (proximal descending and transverse colon) with primary anastomosis and subsequently adjuvant chemotherapy. In 2009, at 63 years of age, he underwent surgery after suspecting recurrence of CRC by laboratory/imaging studies. A rectosigmoid tumorous mass was resected and end ileostomy was placed. Pathologic analysis determined that the mass was a moderately differentiated, mucin-producing adenocarcinoma that invaded through the muscularis propria into the subserosa with two out of three lymph nodes positive for metastasis (T3N1M0). The proband's family history was also positive for colorectal cancer: both his mother (diagnosed at the age of 61 years) and sister were diagnosed with colorectal adenocarcinomas. The proband's sister, a 58-year-old female with history of hypertension, hypercholesterolemia, and chronic smoker (10.5 packs a year), was also diagnosed with colon cancer in 2007 after presenting with weight loss and lower gastrointestinal bleeding. Her colonoscopy showed a small necrotic ulcerated mass at 70 cm in the proximal transverse colon, which was confirmed to be an adenocarcinoma with moderate differentiation extending to the muscularis propria without involving the serosa (T2N1). No evidence of distant metastasis was observed by imaging. A detailed pedigree is shown in Figure 1.

### 2.3. Genetic Analysis

Tumor samples are assigned a unique bar-code for robotic specimen tracking. Commercial immunohistochemistry assays for MMR proteins (MSH2, MLH1, MSH6, and PMS2) and microsatellite instability analysis are performed in all tumor samples by GENZYME [19, 20]. Genetic diagnosis of Lynch syndrome was established by identifying mutations by commercial sequence analyses (MYRIAD Genetic Laboratories, Inc.) [21]. DNA is extracted and purified from peripheral blood samples or buccal mouthwash samples, submitted for molecular testing.

#### 2.3.1. Full Sequence Analysis

Full sequence determination of* MLH1* is performed in both forward and reverse directions of approximately 2,300 base pairs comprising 19 exons and approximately 560 adjacent noncoding intronic base pairs using fluorescent dye-labeled sequencing primers [21]. Aliquots of patient DNA are subjected to polymerase chain reaction (PCR) amplification to generate exon-specific amplicons that can be sequenced directly. Electropherogram tracings of each amplicon are analyzed by a proprietary computer-based review system followed by visual inspection and confirmation of all clinically significant variants [21]. Genetic variants are detected by comparison with a consensus wild-type sequence constructed for each gene. All potential clinically significant variants are independently confirmed by repeated PCR amplification of the indicated gene region(s) and sequence determination as described above.

#### 2.3.2. *MLH1* Large Rearrangement Analysis

Genomic DNA from patients is analyzed by microarray-CGH analysis to determine copy number abnormalities indicative of deletion or duplication mutations across the* MLH1* gene [21]. Approximately 1200 probes have been designed to interrogate all coding exons, limited flanking intron regions, and the respective promoters of* MLH1* and exons 2-3, 8-9. Approximately 220 probes have been designed to interrogate all coding exons. Each probe is analyzed using proprietary software that compares the ratio of bound patient DNA to that of a reference DNA to indicate regions of altered copy number. The microarray design includes probes to detect deletions and duplications in multiple genes tested by MGL; however, a data masking feature is used to limit the analysis only to specific genes for which testing has been requested. Patient samples positive for deletions or duplications are confirmed by repeat microarray analysis of the genes.

#### 2.3.3. Single Site

DNA sequencing analysis is performed for a targeted gene region containing the specified variant in* MLH1.* Microarray-CGH analysis is performed for all single site large rearrangements in* MLH1* [21]. In some cases, long-range PCR analysis and/or sequencing of the resulting PCR product is used to detect specific, previously reported insertions in* MLH1*.

## 3. Results

Our proband's family history pedigree shows the classical Amsterdam's criteria for Lynch syndrome. On the pedigree we show five family members with history of colorectal carcinoma, two with concurrent secondary cancers (breast cancer, lung cancer, and endometrial cancer), and one family member with stomach cancer, which is associated with Lynch syndrome. In the family line the first member to be diagnosed with any type of cancer is the proband's grandmother, diagnosed with stomach cancer at 50 years old. In the second generation, we can acknowledge that all of the offspring were diagnosed with colorectal carcinoma. In the third generation colorectal carcinoma was also diagnosed in three of the four family members including the proband and his sister. A full detailed pedigree is shown in Figure 1.

Commercial immunohistochemistry and microsatellite instability analysis showed absence of MLH1 and PMS2 protein expression and that the tumor was MSI-High. These same analyses were also performed with samples from the proband's sister with similar results (absence of MLH1 and PMS2 protein expression and MSI-High). Genetic sequence analysis detected a novel, frameshift mutation at c.2044_2045 in exon 18 of the* MLH1* gene. A deletion of two consecutive nucleotides (AT) from the positions c.2044 and c.2045 at exon 18 causes a frameshift of the protein coding sequence at p.682 and a premature termination of the MLH1 protein eleven amino acids away from the frameshift site. This same mutation was also found in the proband's sister's daughter and in the proband's son using single site analysis performed by Myriad Genetic Laboratories [21]. These family members remain carriers for mutation since neither of them has shown evidence of tumorous lesion in colon or any other region associated with Lynch syndrome. The proband also presented with a polymorphism in exon 8 of the* MLH1* gene. This variant is caused by an A>G change at the nucleotide 655 (c.655A>G). This single nucleotide substitution leads to the substitution of the isoleucine by a valine at position p.219 in the MLH1 protein. This polymorphism has no known clinical significance.

## 4. Discussion

Lynch syndrome is a highly penetrant, autosomal dominant-susceptibility syndrome that affects less than 5% of all CRC cases [8, 9]. It is associated with germline mutations in MMR genes, which increases risk for colorectal and endometrial cancer, as well as gastric, ovarian, urinary, cutaneous sebaceous glands, and brain cancers [8]. In 1991, the Amsterdam criteria were developed in order to identify families with this autosomal dominantly inherited CRC without a polyposis phenotype. However these criteria have limited sensibility and specificity since 40% of families with known MMR gene mutations do not fulfill the Amsterdam criteria and approximately 50% of families who meet the Amsterdam criteria do not have detectable defect in the MMR genes. The most reliable way to diagnose Lynch syndrome is to detect a mutation in the MMR genes in the suspected patient [2]. To date, there are more than 2,400 different MMR gene mutations described throughout the world (http://www.med.mun.ca/MMRvariants/), most of them in the* MLH1* and* MSH2* genes [22].

Our familial cancer registry, PURIFICAR, contains 35 Hispanic families with MMR mutations or MMR protein deficient tumors. In this registry, we identified our proband, a 63-year-old male diagnosed with CRC at age 42. This patient's family history fulfilled Amsterdam criteria for Lynch syndrome with more than three family members with CRC in two consecutive generations and more than one family member diagnosed earlier than the age of 50 years. Also, the proband's family history presents with multiple cancers highly associated with Lynch syndrome such as endometrial carcinoma and stomach cancer (Figure 1). The compliance with Amsterdam criteria and the incidence of Lynch syndrome-associated cancers within the proband's family further support the Lynch syndrome diagnosis.

**Figure 2:**
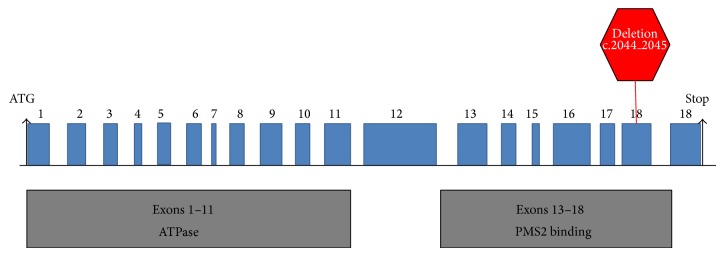
Diagrammatic representation of* hMLH1* gene with novel mutation. The deletion c.2044_2045 mutation at p.682 described causes a premature stop as highlighted in red. Exons are depicted in blue. The regions encoding functional domains are represented by grey boxes [22].

Upon the evaluation of the proband's MMR sequence analysis, we found a deletion at the level c.2044 and c.2045 at exon 18 of the* hMLH1* gene. This deletion was subsequently compared with the available international databases (International Society for Gastrointestinal Hereditary Tumors) [23]; no report of this deletion was found. Therefore, this deletion in* hMLH1* may represent a unique mutation in Puerto Rican Hispanics. The AT at position c.2044 and c.2045 in exon 18 causes a frameshift and subsequently a premature truncation of the MLH1 protein at amino acid 692. It is a deleterious mutation resulting in the malformation of MLH1. A diagrammatic representation of* hMLH1* gene with novel mutation is presented in Figure 2. The newly identified mutation causes a premature STOP codon, leading to a nonfunctional protein, which could not be detected by immunohistochemistry analysis. The single nucleotide polymorphism identified in the proband (substitution A>G at the nucleotide 655 (c.655A>G) of the exon 8 of the* MLH1* gene), however, is of unknown significance and has been described in the literature before [24–27].

The strong history of CRC and Lynch syndrome-associated cancers suggests that the* hMLH1* deletion originated in the maternal line, possibly from the proband's grandmother. However, the origin of the mutation cannot be definitely defined since most of the maternal family line is deceased and genetic testing could not be performed. Though, the de novo mutation seems to be pathogenic since the deletion was present in the subsequent progeny. Similar reports of newly described germline mutations have been described in several ethnic and racial populations [14, 15, 28, 29]. Alvarez et al. described a spectrum of mutations on Chilean families where they also described two unique novel mutations (*MLH1* intron 15 c.1731 +3 A>T/skipping exon 15;* MSH2* exon 13 c.2185_2192delATGTTGGAinsCCCT p.M729_E731delinsP729_X730) associated with strong Amerindian genetic ancestry [30]. Other highly penetrant mutations have been described among Ashkenazi Jews such as the* MSH2* c.1906G/C (p.Ala 636 Pro), which leads mainly to colorectal cancer [31]. Two recently published articles by Dominguez-Valentin et al. discussing the mutation spectrum of South America in Lynch families also showed a high rate of novel mutations in* hMLH1* and* hMSH2* [32, 33]; however neither article presented the mutation found in our patient.

To confirm the presence of Lynch syndrome, the MMR genes of the individuals with MSI tumors are screened for germline MMR mutations. Similar to the other MMR genes, diverse sequence variants, including nonsense, missense, splice site, and frameshift mutations, have been reported throughout the coding region of the* MLH1* gene [34]. In Puerto Rico, a population-based study conducted by de Jesus-Monge et al. [35] reported that the prevalence of deficient MMR protein expression (MLH1 and MSH2) among CRC patients was 4.3% with most cases having absence of MSH2 proteins. This study highlights the importance of genetic testing for Lynch syndrome in our Hispanic population and the need to increase our current understanding of common mutations and reducing variables of uncertain significance (VUS). VUS are mainly missense substitutions that result in single amino acid changes but also include in-frame small deletions or insertions that change only small numbers of amino acids, silent coding alterations that may influence splicing or translation or intronic changes of unknown influence on gene splicing. VUS are usually classified as either “pathogenic” or “not pathogenic/low clinical significance” [36]. About 24% of mutations detected in* MLH1* are missense substitutions; the majority of these are referred to as unclassified genetic variants [34]. Given that the diagnosis of Lynch syndrome relies primarily on the identification of germline defects in the MMR genes, detecting these unclassified genetic variants creates ambiguity in the clinical setting, as the pathogenicity of these variants cannot be readily ascertained [34]. Clinical implications of missense, silent variants, or mutations close to splice site are often not clear. Therefore, those variants are classified as VUS and represent up to 30% of the identified DNA changes in MMR genes [37]. Currently the International Society Collaborative Research for Gastrointestinal Hereditary Tumors (InSight) is trying to characterize all the missense, deletions, insertions, or any other mutation that may affect MMR genes with the aim of distinguishing nonpathogenic from pathogenic mutations.

To date, there is very limited information about the prevalence of MSI among Hispanic CRC patients. Gupta et al. reported in a low prevalence of MSI in the majority studies including Hispanics with CRC, which suggests that MSI status likely results from germline genetic defects as seen in Lynch syndrome [2]. This finding emphasizes the importance of genetic testing in the Hispanic community in order to identify individuals with Lynch syndrome. Our report of a novel mutation in* hMLH1* contributes to the characterization of pathogenic mutations in the MMR genes leading to Lynch syndrome.

## Core Tip

Very little is known about the prevalence, clinical manifestations, and genetic mutations in US Hispanic individuals with hereditary CRC. Hereditary nonpolyposis colorectal cancer** (**HNPCC), otherwise known as Lynch syndrome, has been described in up to 5% of CRC cases. This case report presents a unique mutation in the MLH1 gene in a Hispanic family from Puerto Rico leading to a classic case of Lynch syndrome. Our aim is to present this novel mutation to the medical community.
